# Exploring the Cold-Adaptation Mechanism of Serine Hydroxymethyltransferase by Comparative Molecular Dynamics Simulations

**DOI:** 10.3390/ijms22041781

**Published:** 2021-02-11

**Authors:** Zhi-Bi Zhang, Yuan-Ling Xia, Guang-Heng Dong, Yun-Xin Fu, Shu-Qun Liu

**Affiliations:** 1 State Key Laboratory for Conservation and Utilization of Bio-Resources in Yunnan & School of Life Sciences, Yunnan University, Kunming 650091, China; zhangzhibi@kmmu.edu.cn (Z.-B.Z.); xiayl@ynu.edu.cn (Y.-L.X.); dgh@mail.ynu.edu.cn (G.-H.D.); 2 Yunnan Key Laboratory of Stem Cell and Regenerative Medicine & Biomedical Engineering Research Center, Kunming Medical University, Kunming 650500, China; 3 Human Genetics Center and Division of Biostatistics, School of Public Health, The University of Texas Health Science Center, Houston, TX 77030, USA

**Keywords:** cold adaptation, molecular dynamics simulation, stability-flexibility-activity relationships, protein-solvent interactions, free energy landscape

## Abstract

Cold-adapted enzymes feature a lower thermostability and higher catalytic activity compared to their warm-active homologues, which are considered as a consequence of increased flexibility of their molecular structures. The complexity of the (thermo)stability-flexibility-activity relationship makes it difficult to define the strategies and formulate a general theory for enzyme cold adaptation. Here, the psychrophilic serine hydroxymethyltransferase (pSHMT) from *Psychromonas ingrahamii* and its mesophilic counterpart, mSHMT from *Escherichia coli*, were subjected to μs-scale multiple-replica molecular dynamics (MD) simulations to explore the cold-adaptation mechanism of the dimeric SHMT. The comparative analyses of MD trajectories reveal that pSHMT exhibits larger structural fluctuations and inter-monomer positional movements, a higher global flexibility, and considerably enhanced local flexibility involving the surface loops and active sites. The largest-amplitude motion mode of pSHMT describes the trends of inter-monomer dissociation and enlargement of the active-site cavity, whereas that of mSHMT characterizes the opposite trends. Based on the comparison of the calculated structural parameters and constructed free energy landscapes (FELs) between the two enzymes, we discuss in-depth the physicochemical principles underlying the stability-flexibility-activity relationships and conclude that (i) pSHMT adopts the global-flexibility mechanism to adapt to the cold environment and, (ii) optimizing the protein-solvent interactions and loosening the inter-monomer association are the main strategies for pSHMT to enhance its flexibility.

## 1. Introduction

More than three-quarters of the Earth’s surface is occupied by cold ecosystems, including the Arctic, Antarctic, Alpine regions and deep seas. Despite the poor living conditions provided by the cold environment, cold-adapted organisms, also called psychrophiles, have evolved to live in such harsh conditions. Temperature is one of the most important environmental factors in biological metabolism because the enzymatic reactions closely associated with life activities could be reduced by 30–80 folds as the temperature decreases from 37 to 0 °C [1]. Therefore, the main challenge confronted by the psychrophiles is how to maintain a sufficiently high enzymatic reaction rate/catalytic activity at low temperatures [2].

The psychrophilic (or cold-adapted) enzymes, which refer to those produced by psychrophiles, are characterized by a lower thermostability and higher catalytic activity at low temperatures when compared to their mesophilic (warm-active) or thermophilic (heat-adapted) homologues [3]. The low thermostability of cold-adapted enzymes should be a consequence of weakening intra-molecular forces, which in turn leads to the enhanced conformational dynamics or flexibility, thus making it easy to accomplish the conformational changes required for catalysis at low temperatures. Therefore, it appears that the flexibility of the molecular structure establishes a link between the thermostability and catalytic activity of enzymes, with increased flexibility of the psychrophilic enzyme not only lowering the thermostability (or structural stability) but also maintaining a high catalytic activity at low temperatures [4]. However, the actual (thermo)stability-flexibility-activation relationships seem to be more complicated than the above description would suggest.

First, although the structural basis of the decreased thermostability of a psychrophilic enzyme is its increased conformational flexibility, the maintenance of the structural integrity, which is necessary for the enzyme function, is dictated by a delicate balance between the flexibility and rigidity involving different structural regions of the psychrophilic enzyme. Second, although a psychrophilic enzyme could evolve towards the lowest possible stability of its native state due to the strong selection pressure for the catalytic activity at low temperatures [5], it is difficult to distinguish between the structural regions of increased flexibility that are associated with increased activity or with decreased thermostability. Third, although differently temperature-adapted members within the same enzyme family share highly similar 3D structures, the protein structure is complicated, with the topological organization varying considerably across different families; therefore, the molecular determinants and/or structural factors that dictate the differences in the flexibility/rigidity between differently temperature-adapted members can change in different enzyme families, thus making it difficult to define the cold-adaptation strategies and to formulate a general or unified theory for enzyme cold adaptation [4].

High-resolution X-ray crystallographic structures have provided invaluable insights into the structure-function relationships of proteins; however, these static pictures provide very limited information about the dynamic and thermodynamic nature of proteins, which is crucial to understanding the molecular mechanisms that rule the stability-flexibility-activity relationships of the psychrophilic enzymes. Moreover, because the fluctuations of single atoms are difficult to measure in real time, it is a challenging task using the experimental techniques to compare the differences in the dynamics (including the structural fluctuations, conformational flexibility, and molecular motions) and thermodynamics (i.e., relative populations of different states/substates) between differently temperature-adapted homologous enzymes within the same family [4,6]. Thanks to the molecular dynamics (MD) simulation technique, this kind of computational approach can provide information about the precise position of each atom within a protein structure as a function of the simulation time, based on which the conformational flexibility, molecular motion modes, and a representation of the free energy landscape (FEL) near the protein native state can be derived [7,8,9]. Therefore, in this paper, we employed the MD simulations to explore the cold-adaptation mechanism of serine hydroxymethyltransferase (SHMT; EC 2.1.2.1).

**Figure 1:**
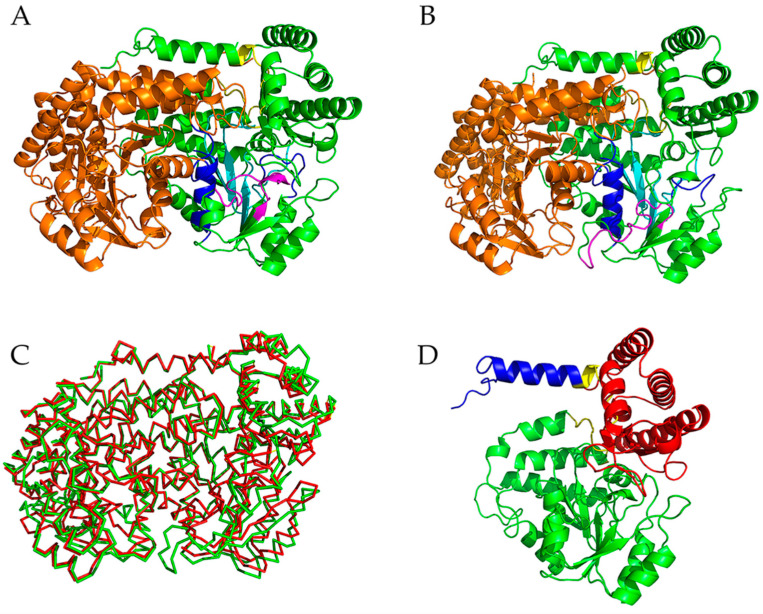
Cartoon representations of the crystal structures of two differently temperature-adapted serine hydroxymethyltransferases (SHMT) and their backbone superposition. (**A**,**B**) The dimeric forms of the mesophilic SHMT (mSHMT) from *Escherichia coli* (PDB ID: 1DFO [18]) and the psychrophilic SHMT (pSHMT) from *Psychromonas ingrahamii* (PDB ID: 4P3M [19]), respectively. (**C**) Backbone superposition of the two structures. (**D**) The monomeric form of mSHMT. The missing residues in the crystal structures were modeled as described in Section 4.1. In (**A**,**B**), the monomer-A and monomer-B are colored green and orange, respectively; the active-site components of the monomer-A, i.e., the “floor”, “walls”, and “roof” are colored cyan, blue, and magenta, respectively. In (**C**), the backbones of mSHMT and pSHMT are colored red and green, respectively. In (**D**), The N-terminal arm, large domain, small domain, and inter-domain linkers are colored blue, green, red, and yellow, respectively.

SHMT, which belongs to the aspartate aminotransferase superfamily (fold type I), is a pyridoxal phosphate (PLP; vitamin B6)-dependent enzyme [10]. Using PLP as the cofactor, SHMT carries out interconversion of serine and glycine by catalyzing the reversible transfer of C_β_ of serine to tetrahydropteroylglutamate (H_4_PteGlu), resulting in the formation of glycine and 5,10-methylene-H_4_PteGlu [11,12]. This reaction provides a primary source of one-carbon units required for the synthesis of thymidylate, purines, and methionine [13]. SHMT also catalyzes the H_4_PteGlu-independent cleavage of many 3-hydroxyamino acids and decarboxylation of aminomalonate, which is similar to H_4_PteGlu-dependent serine cleavage [14]. Because of the observed increased activity of SHMT in neoplastic tissues and its essential role in nucleotide biosynthesis, SHMT has been suggested as a potential target for cancer therapy [15,16]. In prokaryotes, the catalytically functional form of SHMT is a dimer, whereas in eukaryotic cells, the active enzyme exists as a tetramer [11]. It has been shown that the catalytic activity of the cold-adapted SHMT from the psychrophilic bacterium *Psychromonas ingrahamii* (pSHMT) is at least ten-fold higher than that of the mesophilic *Escherichia coli* SHMT (mSHMT) in the temperature range of 10–50 °C, and the thermostability, measured as the T_m_ value, is lower for pSHMT than for mSHMT, in particular at the non-functional monomer level [17]. Nevertheless, these two enzymes share an amino acid sequence identity as high as 75% (ESI Figure S1) and a very similar 3D architecture (Figure 1), with the C_α_ root mean square deviation (RMSD) value between the determined crystal structures [18,19] of 0.83 Å.

Although a previous study [20] performed using the comparative analyses of the amino acid residue properties and conformational flexibility among the three temperature populations of SHMTs (psychrophilic, mesophilic, hyper- and thermophilic) suggests that the cold adaptation of SHMT has been achieved through general increases of polarity and flexibility at the protein core, surface, and interface, the structures used in this study were built using the homology modeling technique (no experimental structure of any psychrophilic SHMT was available at that time) and no explicit data about the flexibility was provided. Furthermore, a simple comparison between the static, very similar crystal structures of pSHMT [19] and mSHMT [18] cannot provide explicit information about the differences in the dynamics and thermodynamics between the two enzymes. Thus, here we performed μs-scale multiple-replica MD simulations on the dimeric forms of the two enzymes to investigate these differences and, further, to attempt a better understanding of the physicochemical principles underlying the relationships between the thermal/structural stability, conformational dynamics/flexibility, and functional properties of SHMTs. Our results not only uncover the cold-adaptation strategies but also shed light on the mechanism for pSHMT to adapt to low temperatures.

## 2. Results

### 2.1. Descriptions of the Crystal Structures of mSHMT and pSHMT

Figure 1 shows the crystal structures of mSHMT and pSHMT with modeled missing residues. The two monomers (monomer-A and -B) in a functional dimer have almost identical structures and associate with each other in a mirror-like arrangement (Figure 1A,B). The structure of the monomeric form can be divided into three domains (Figure 1D): a N-terminal arm (blue), a large domain (green), and a small domain (red). The N-terminal arm, which is mainly composed of α-helices, wraps around the other monomer in the functional dimer and participates in maintaining association between the two monomers. The large domain contains the PLP binding site and is characterized by an αβα fold with a seven-stranded mixed β-sheet surrounded by α-helices on both sides. The small C-terminal domain folds into an α/β sandwich consisting of a four-stranded antiparallel β-sheet and five α-helices. The functional dimer contains two active sites, each in one monomer. Of note is that the entrances of both active-site cavities are located at the inter-monomer interfaces, and some of the residues participating in the formation of the active site in one monomer come from the other monomer [16,19].

### 2.2. Structural Fluctuations during Simulations and Conformational Sampling Evaluation

**Figure 2:**
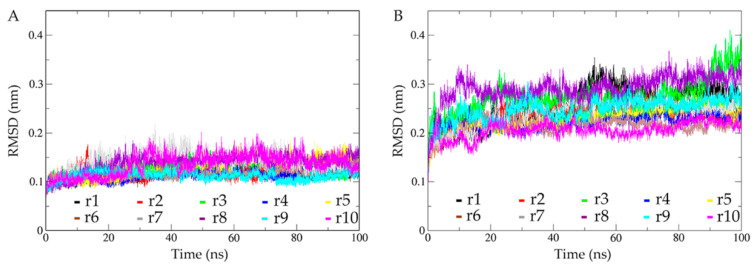
Time evolution of the C_α_ root mean square deviation (RMSD) values of mSHMT and pSHMT with respect to their respective starting structures calculated from the 10 MD simulation replicas (r1-10). (**A**) mSHMT. (**B**) pSHMT.

The structural fluctuations during the MD simulations were assessed by computing the time evolution of C_α_ RMSD with respect to the starting structure (Figure 2). As shown in Figure 2A, all 10 replicas of the dimeric mSHMT require only a few ps to reach relatively stable RMSD values. For the dimeric pSHMT (Figure 2B), although most of the replicas have reached an equilibrium within a few ps, some replicas exhibit a continuous increase in RMSD values until after about 10 ns. For purpose of the temporal consistency of the two simulation systems, we arbitrarily took the 10–100 ns trajectory as the equilibrated portion of each replica. It is clear that the equilibrated portions of the 10 replicas have a narrower RMSD range for mSHMT (0.07–0.20 nm) than for pSHMT (0.15–0.40 nm), and that the RMSD fluctuation amplitudes of most replicas are smaller for mSHMT than for pSHMT, implying the enhanced conformational dynamics of pSHMT. To quantitatively evaluate whether pSHMT experienced enhanced structural fluctuations compared to mSHMT, the standard deviations (SDs) of the RMSD means were calculated from the equilibrated portions of the respective 10 replicas for the two systems, and the one-sided *t*-test was performed on the two sets of SDs (ESI Table S1). The obtained p-value of 0.014 indicates that pSHMT simulations produced a statistically significantly higher SD of the RMSD mean than mSHMT simulations, confirming that pSHMT experienced larger global structural fluctuations than mSHMT.

In order to further evaluate the structural fluctuations of the two individual monomers and their relative mobility with respect to each other, we calculated the time-dependent C_α_ RMSD of one monomer by least-squares fitting to the same monomer (self-fitting) and to another monomer (non-self-fitting) in the starting dimeric structure, respectively (ESI Figure S2). As expected, for both pSHMT and mSHMT, their individual monomers show higher RMSD fluctuation amplitudes when using the non-self-fitting approach than when using the self-fitting approach. However, no matter which fitting approach was employed, the individual monomers of pSHMT show a wider RMSD range and higher RMSD fluctuation amplitudes than those of mSHMT, in particular when using the non-self-fitting approach. These observations indicate that, for the two monomers in the context of the functional dimer: (i) they themselves experienced larger structural fluctuations in pSHMT than in mSHMT, and (ii) they experienced larger relative position shifts in pSHMT than in mSHMT.

Taken together, our RMSD calculations reveal that, when compared to the warm-active mSHMT, the cold-adapted pSHMT experienced not only more drastic conformational fluctuations at both the levels of the entire dimer and individual monomers, but also larger relative monomer movements. Thus, pSHMT has a lower structural stability and a stronger capability to change conformation than mSHMT.

In the multiple-replica MD simulations, different replicas can sample different directions around the starting structure and hence improve the sampling ergodicity in the conformational space [21]; therefore, this type of simulation strategy has often been employed to enhance the sampling efficiency of the protein conformational space [8,9,22,23]. For the 10 independent replicas of each simulation system, their first 10-ns trajectories were discarded and the remaining portions (10–100 ns) were concatenated into a single joined 900-ns equilibrium trajectory. The cosine contents of the first two eigenvectors obtained from the essential dynamics (ED) analyses of the individual replicas and the single joined trajectories were calculated to evaluate the degree of sampling convergence. The results (ESI Table S1) show that, for both systems, the concatenation of the individual replicas effectively lowers the cosine contents when compared to many individual replicas, indicating that the single joined trajectories achieve a relatively higher degree of sampling convergence compared to a single replica. Therefore, all the subsequent analyses were performed based on the single joined trajectories of mSHMT and pSHMT to ensure that the calculated parameters reflect the intrinsic properties of these two enzymes.

### 2.3. Comparison of Structural Properties

In order to compare the structural properties of mSHMT and pSHMT, the average values of several structural/geometrical parameters, i.e., the solvent accessible surface area (SASA), the number of close inter-atomic contacts (NCIC), radius of gyration (Rg), and the number of hydrogen bonds (NHB), were calculated from the joined equilibrium trajectories (Table 1). As listed in Table 1, pSHMT has lower average values of NCIC and intra-protein NHB while higher average values of SASA, Rg, and protein-solvent NHB than pSHMT; furthermore, with the exception of the protein-solvent NHB, all the parameters have higher SDs for pSHMT than for mSHMT. For pSHMT, the decreased NCIC and intra-protein NHB, accompanied by their increased variability (reflected by SDs), imply the weakening of the intra-molecular forces that stabilize the protein structure, thus explaining the decreased overall structural stability of pSHMT compared to mSHMT; the increased protein-solvent NHB, together with its decreased variability, implies the strengthening of the favorable interactions of the protein with water, which has been suggested to facilitate conformational fluctuations of the protein structure [20,22,24]; the increased SASA and Rg mean a less compact packing and a larger size of the structure of pSHMT, respectively, compared to the structure of mSHMT.

In general, the above analyzed properties are intrinsically related with one another: the strengthened interactions between the protein and solvent could facilitate the weakening of the intra-protein forces, which in turn is conducive to the conformational changes/fluctuations and hence leads to the relaxation of the protein structure.

### 2.4. Comparison of Conformational Flexibility

**Figure 3:**
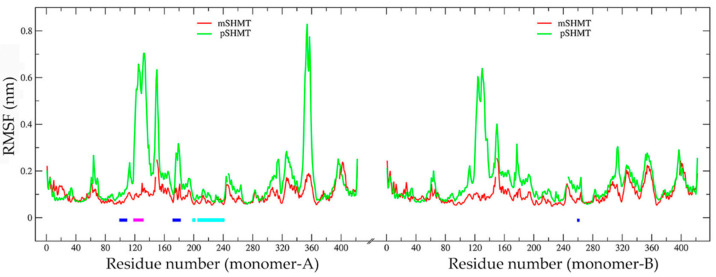
Per-residue C_α_ Root-mean-square fluctuation (RMSF) profiles of mSHMT (red line) and pSHMT (green line). Residues that constitute the “floor” (residues 197–204 and 213–242), “walls” (inner “wall”: residues 97–110 from the monomer-A and 258–264 from the monomer-B; outer “wall”: residues 174–182 from the monomer-A), and “roof” (residues 118–133) of the active site in the monomer-A are indicated above the horizontal axis by line segments colored in cyan, blue, and magenta, respectively.

Figure 3 shows the C_α_ RMSF profiles of the two enzymes as a function of the residue number, which is according to the mSHMT crystal structure (ESI Figure S1). As shown in Figure 3, the two monomers (i.e., monomer-A and -B) within a functional dimer (pSHMT or mSHMT) exhibit very similar RMSF profiles. This is not surprising, as the two monomers within the same dimeric enzyme have almost identical 3D structures and contact interfaces. However, despite the similar changing trends of the RMSF profiles for the same monomers (monomer-A or -B) from different enzymes, the monomers from pSHMT exhibit, in almost all structural regions, higher RMSF values, and in particular in several surface loop regions (i.e., residues 119–133, 146–153, and 174–181 in both monomers and 349–359 in the monomer-A) considerably higher RMSF values than the corresponding ones from mSHMT. These observations, together with a higher RMSF average value of pSHMT (0.16 ± 0.12 nm) than that of mSHMT (0.10 ± 0.04 nm), indicate a higher global conformational flexibility of pSHMT. Nevertheless, there are still few regions that exhibit slightly lower RMSF values in pSHMT than in mSHMT, i.e., residues 9–28 in the N-terminal arms of the both monomers, residues 38–52 in the large domain of the monomer-B, and residues 393–405 in the small domain of the monomer-A. Of note is that the former two regions are involved in the direct contacts between the monomer-A and -B and participate in the formation of the inter-monomer interfaces, for which most of the constituting regions have higher RMSF values in pSHMT than in mSHMT.

In SHMT, each of the two monomers contains an active site, with its “floor” formed by β6 (residues 197–202), β7 (residues 223–228), β8 (residues 237–242), and the loops (residues 203–204, 213–222, and 229–236) connecting these β-strands, the inner “wall” formed by α6 (residues 97–110) and a loop (residues 258–264 from the other monomer), the outer “wall” formed by a loop of residues 174–182, and the “roof” by the loop spanning residues 118–133 [19]. Close inspection of Figure 3 reveals that all the active-site forming segments of both monomers have higher RMSF values in pSHMT than in mSHMT. The calculated RMSF average values for the active-site “floor”, “walls”, and “roof” are listed in Table 2. As shown in Table 2, the “floor” of each active site has higher flexibility in pSHMT than in mSHMT, while the “walls” and “roof” of each active site have considerably higher flexibility in pSHMT than in mSHMT.

In the dimeric SHMT, each monomer contains a PLP-binding site and a H_4_PteGlu-binding site. Since these cofactor sites are located at the interfaces between the two monomers, their constituting residues come from both monomers. For example, the PLP site in the monomer-A is formed by residues Ser35, Gly98, Ser99, His126, Ser175, Asp200, His203, Thr226, His228, Lys229, Arg235, and Arg363 from the monomer-A, and Tyr55, Glu57, Tyr65, and Gly263 from the monomer-B [18]. Almost all the PLP-site residues have higher RMSF values in pSHMT than in mSHMT, with the average values in the monomer-A of 0.142 ± 0.137 and 0.079 ± 0.015 nm, respectively, and in the monomer-B of 0.124 ± 0.108 and 0.089 ± 0.020 nm, respectively. The H_4_PteGlu site is composed of residues Leu121, Gly125, Leu127, and Asn347 from one monomer and residues Glu57 and Tyr64 from the other monomer [18], all of which have higher RMSF values in pSHMT than in mSHMT, with the average values in the monomer-A of 0.377 ± 0.238 and 0.094 ± 0.010 nm, respectively, and in the monomer-B of 0.330 ± 0.191 and 0.099 ± 0.023 nm, respectively. Note that the PLP-site residues His126 and Ser175 and the H_4_PteGlu-site residues Leu121, Gly125, and Leu127 are located in the loops that exhibit considerably higher flexibility in pSHMT than in mSHMT.

In summary, the cold-adapted pSHMT is characterized not only by a higher global flexibility but also by considerably higher local flexibilities of the active sites and cofactor sites as compared to mSHMT in MD simulations.

### 2.5. Essential Dynamics and Collective Motions

**Figure 4:**
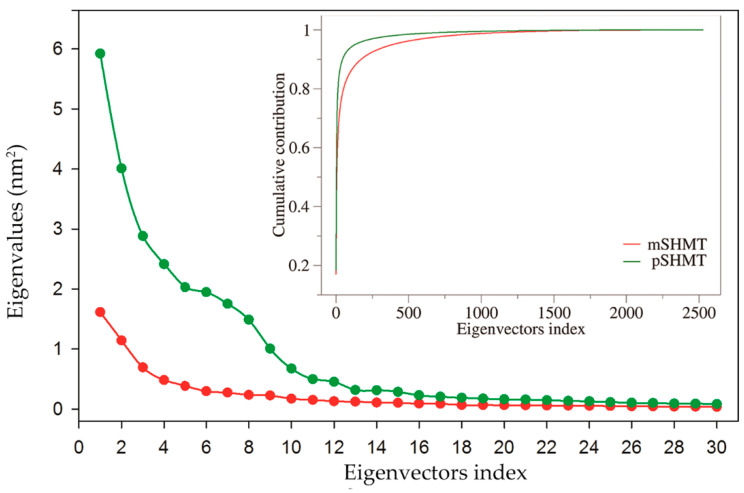
Eigenvalues of the first 30 eigenvectors (main plot) and cumulative contribution of all eigenvectors to the total mean square fluctuations (inset plot) obtained from essential dynamics analyses of the single joined equilibrium MD trajectories of mSHMT (red line) and pSHMT (green line).

ED analysis was performed on the joined equilibrium trajectories to investigate the difference in the largest-amplitude collective motions between mSHMT and pSHMT. The total mean square fluctuation (TMSF) values obtained after the diagonalization of the C_α_ covariance matrices for mSHMT and pSHMT are 9.47 and 32.29 nm^2^, respectively, indicating that pSHMT experienced considerably larger atomic fluctuations than mSHMT during simulations, in agreement with the above comparative analyses in terms of RMSD and RMSF. Figure 4 shows the eigenvalues of the first 30 eigenvectors and the cumulative contribution of all eigenvectors to TMSF. It is clear that the eigenvalues of both enzymes and the differences between them decrease with increasing the eigenvector index. However, the first 10 eigenvectors of pSHMT have significantly higher eigenvalues than the corresponding ones of mSHMT, indicating larger fluctuation amplitudes (or more dramatic conformational changes) of pSHMT along these eigenvectors. Moreover, for mSHMT, the cumulative contributions of the first two and 10 eigenvectors to TMSF are 29.1% and 58.3%, respectively, and for pSHMT, the corresponding contribution rates are 30.8% and 74.7%, respectively (Figure 4, inset). Considering the huge conformational space spanned by 3′N eigenvectors (N is the number of C_α_ atoms; 834 and 842 for mSHMT and pSHMT, respectively), it is reasonable to believe that the first 10 eigenvectors, in particular the first two ones, make a substantial contribution to the overall conformational freedom in the space and, therefore, the first two eigenvectors span an essential subspace within which the largest collective motions take place.

**Figure 5:**
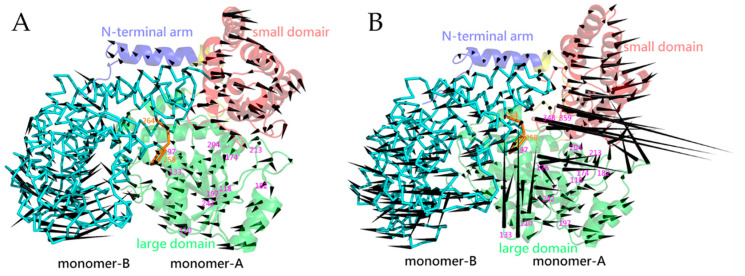
Porcupine plots showing the largest-amplitude collective motion along the first eigenvector. (**A**,**B**) The most significant motion modes of mSHMT and pSHMT, respectively. In a dimer, the monomer-A is rendered in cartoon representation, with the N-terminal arm, large domain, and small domain colored blue, green, and red, respectively; the monomer-B is rendered in ribbon and colored cyan. In the monomer-B, a small segment (residues 258–264) that participates in the formation of the inner “wall” of monomer-A’s active-site cavity is colored orange. In the monomer-A, the residue numbers for the starting and ending residues of the segments participating in the formation of the active-site cavity are labeled.

Since the first eigenvector has the largest eigenvalue, it represents the largest-amplitude collective motion or the most significant motion mode of the protein structure. Figure 5 shows the largest-amplitude motion modes of mSHMT and pSHMT in terms of the porcupine plot, where the pointing direction and length of a cone represent the movement direction and amplitude of a C_α_ atom, respectively. It is clear from this figure that the two monomers of pSHMT (Figure 5B) exhibit larger fluctuation amplitudes than those of mSHMT (Figure 5A), in particular in the small domain, peripheral loops, and the two loops (residues 118–133 and 348–359) in the proximity of the inter-monomer interfaces. Furthermore, although the monomer-A and -B within the same dimer exhibit similar motion modes with respect to each other, the differences in their relative fluctuation directions between mSHMT and pSHMT lead to different conformational consequences of the dimers. For the two monomers within the dimeric mSHMT, their N-terminal arms and small domains move in the directions away from the contact interfaces, while the large domains move toward each other, thus resulting in the widening/loosening of the upper part while the contraction/tightening of the lower part of the dimer (Figure 5A). In the case of the dimeric pSHMT, the small domains and much of the large domains collectively move in the directions away from the inter-monomer interfaces, thus resulting in the overall loosening of the dimer, although the downward movement of the N-terminal arms and the upward movement of a partial base of the large domain in the monomer-A contribute to reinforcing the dimer (Figure 5B).

Close inspection of the segments that form the active-site cavity in the monomer-A reveals that, for both mSHMT and pSHMT, their cavity “floors” (residues 197–204 and 213–242) have a small fluctuation amplitude, in consistence with their low RMSF values as observed in Figure 3; nevertheless, the “floor” exhibits an inward and an outward movement relative to the cavities of mSHMT and pSHMT, respectively, thus contributing to shrinking and enlarging their respective cavities. Furthermore, for mSHMT, although all the segments that form the cavity “walls” (residues 97–110 and 174–182 from monomer A and 258–264 from monomer B) and “roof” (residues 118–133) move collectively toward the inter-monomer interfaces, the larger displacement amplitude of the outer “wall” segment (residues 174–182) contributes to shrinking the cavity. For pSHMT, the “wall”- and “roof”-forming segments exhibit versatile fluctuation directions with the fluctuation amplitude varying drastically. For example, one inner “wall”-forming segment (residues 97–110) moves with a small amplitude toward the cavity, the other inner “wall”-forming segment (residues 258–264) and the outer “wall” segment (residues 174–182) move collectively with a larger amplitude away from the cavity, and the “roof” (residues 118–133), which has the largest fluctuation amplitude, exhibit a twisting-like motion with its N-terminal part (residues 118–126) shifting away from the cavity while the C-terminal part (residues 127–133) shifting toward the interfaces. As a result, despite the complicated collective fluctuations of the cavity-forming segments, their combined effect is to enlarge the active-site cavity of pSHMT. Also worth noting is that the loop (residues 348–359) in the small domain of pSHMT, which resides near the inter-monomer interfaces and cover the entrance of the active-site cavity, moves with the largest amplitude away from the entrance.

### 2.6. Free Energy Landscapes

**Figure 6:**
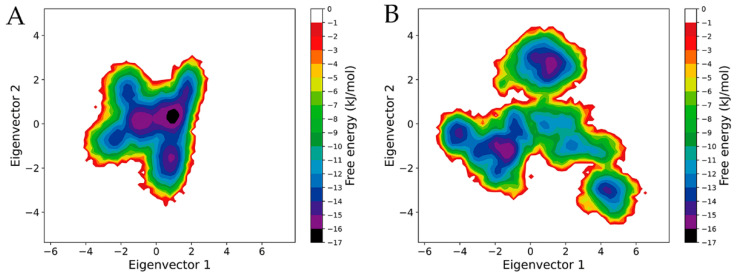
Constructed free energy landscapes (FELs) of mSHMT and pSHMT using the reaction coordinates as the projection of the joined equilibrium MD trajectory onto an essential subspace spanned by eigenvectors 1 and 2. (**A**) FEL of the mSHMT. (**B**) FEL of the pSHMT. The color bar represents the relative free energy value in kJ/mol.

To investigate the differences in the thermodynamics between mSHMT and pSHMT, their FELs were constructed using the probability density function with the reaction coordinates as the projection of the joined equilibrium trajectory onto the first two eigenvectors (Figure 6). It is clear that, when compared to the FEL of mSHMT (Figure 6A), the FEL of pSHMT (Figure 6B) presents a more complex, divergent shape and spans larger ranges along eigenvectors 1 and 2, implying that pSHMT has a more complex kinetic behavior and a larger conformational entropy than mSHMT. Furthermore, the FEL of pSHMT features a more rugged/rough surface due to a greater number of free energy basins/minima compared to that of mSHMT. Specifically, in the FEL of mSHMT, there is only one single, large, continuous minimum free energy basin with an energy level lower than −10 kJ/mol, whereas there are four basins with the same energy level in the FEL of pSHMT; therefore, it can be considered that mSHMT and pSHMT sampled one and four conformational states, respectively, during the MD simulations. As the free energy decreases, the basin in the FEL of mSHMT, although with reduced size, is still continuous until reaching the energy level of −14 kJ/mol, below which two minima can be observed. On the contrary, more minima can be found in the FEL of pSHMT with decreased free energy, e.g., five, four, and four basins/minima at the energy levels lower than −12, −13, and −14 kJ/mol, respectively. Furthermore, the discrete distribution of minima in the FEL of pSHMT implies that there are relatively large conformational differences among the substates of pSHMT. Of note is that, although the FELs of both enzymes contain two global free energy minima (i.e., with an energy level lower than −15 kJ/mol), one of the global minima in the FEL of mSHMT has a larger size and contains a minimum with a lower free energy value (−16 kJ/mol) compared to the two ones in the FEL of pSHMT, indicating that the main substate of mSHMT has a larger population and higher thermal stability than the main substates of pSHMT.

To sum up, pSHMT has a larger conformational entropy, richer conformational diversity, and lower thermostability than mSHMT, in agreement with the above observations that pSHMT has a higher global conformational flexibility/mobility, stronger conformational change ability, and lower structural stability.

## 3. Discussion

In this study, the μs-scale multiple-replica MD simulations of the psychrophilic pSHMT and mesophilic mSHMT were performed to investigate the differences in the dynamics and thermodynamics between them, whereby to explore the cold-adaptation mechanism of SHMT from the perspective of the stability-flexibility-activity relationships. The comparative analyses of the single joined equilibrium trajectories in terms of RMSD and ED reveal that, when compared to mSHMT, pSHMT experienced significantly enhanced global structural fluctuations and more drastic overall conformational changes during simulations, thus indicating a lower structural stability and stronger conformational change ability of pSHMT. Further comparative analysis of RMSF profiles reveals that, with the exception of very limited structural regions that exhibit only slightly higher RMSF values in mSHMT than in pSHMT, the entire structure exhibits higher flexibility in pSHMT than in mSHMT. Our results are in agreement with a previous study suggesting that pSHMT achieves its cold adaptation through an increase in flexibility at the protein core, surface, and interfaces [20]; therefore, we propose that pSHMT likely adopts the global-flexibility mechanism [5,25], rather than the local rigidity/flexibility mechanism [1,4,26], to adapt to the cold environment.

In the global-flexibility mechanism, the enzyme could have evolved toward the highest possible flexibility of the entire structure and, hence, the lowest possible thermostability of its native state. A typical example that exploits this mechanism is the cold-adapted α-amylase [5,27]. In the local rigidity/flexibility mechanism, although a cold-adapted enzyme has acquired a higher flexibility in some structural regions compared to its warm-active counterparts, there are still certain specific regions that have a higher rigidity, thus resulting in the appearance of the domains/substructures characterized by distinctly different thermostabilities [28]. It appears that many of the psychrophilic enzymes studied so far adopt the local rigidity/flexibility mechanism [4,29,30,31]. More interesting examples are the psychrophilic superoxide dismutases, which were shown to possess restricted flexible regions sufficient for low temperature catalysis but not enough extended or mobile to impair the thermostability of them [32]. When compared to the warm- or heat-adapted counterparts, the increased flexibility of a cold-adapted enzyme can contribute to lowering its thermal/structural stability; however, the increased flexibility in the regions that are irrelevant to the catalytic process does not make a positive contribution to the catalytic activity but can impair the activity of the cold-adapted enzyme [1]. The energetic basis underlying the increased catalytic activity of the cold-adapted enzyme is its lower activation free energy compared to the warm-active counterparts, which is fundamentally determined by a considerable reduction in the activation enthalpy [33,34,35] originating from the increased flexibility in the regions involved in the catalytic motions of the cold-adapted enzyme [1]. On the contrary, the increased flexibility in the regions that are not directly involved in the catalytic movements can reduce the activation entropy of the cold-adapted enzyme (i.e., becoming more negative or less positive compared to the activation entropy of its warm-active counterparts), which in turn counteracts the positive effect of the reduced activation enthalpy relative to the warm-active counterparts, thus reducing the difference in the activation free energy between the cold- and warm-active enzymes and impairing the gain in the catalytic activity; while the increased rigidity in the regions not directly involved in the catalytic motions can alleviate the negative effect of the reduced activation entropy [1].

Despite the advantage of the local rigidity/flexibility in gaining the catalytic activity, our MD simulation results suggest that pSHMT is unlikely to adopt the local rigidity/flexibility mechanism. Undoubtedly, the observed increase in flexibility of almost the entire structure of pSHMT explains its reduced thermostability compared to mSHMT. Although it has been suggested that the increased flexibility in the regions that are involved in the catalytically relevant motions can reduce the activation enthalpy, such regions in pSHMT still remain to be identified in future work. Interestingly, it has been found that the increased protein surface flexibility in several cold-adapted enzymes is directly related to the reduced activation enthalpy compared to the warm-active counterparts [34,36,37], implying that the surface mobility could act to modulate the conformational changes occurring during catalysis through the interaction network and correlated motions [38,39,40]. For pSHMT, it is possible that the positive effect (i.e., reduced activation enthalpy) arising from the considerably increased flexibility in the surface loops (Figure 3) could over-counteract the negative effect (i.e., reduced activation entropy) arising from the increased flexibility in the regions not involved in the catalytic motions, thus resulting in a lower activation free energy and explaining its increased catalytic activity compared to mSHMT. 

The calculated structural/geometrical parameters (Table 1) indicate that pSHMT has a less compact structural packing and a larger molecular size, while fewer and weaker intra-molecular interactions. This is not surprising, as the weakening of intra-molecular interactions/forces will cause the relaxation of the molecular structure. A more important observation is that pSHMT strengthens its hydrogen-bonding interactions with the solvent compared to mSHMT. In our previous studies, we have demonstrated that the intensified protein-solvent hydrogen-bonding interactions can transfer efficiently the solvent kinetic energy to the protein surface and hence greatly facilitate fluctuations of the solvent-exposed loops that lack the conformational constraints [22]; furthermore, such enhanced loop mobility can transmit via the specific mechanic mechanisms (e.g., hinge-bending mechanism and neighborhood effect) and the network of intra-molecular interactions over the entire structure, thus leading to the increased flexibility and enhanced collective motions of the protein structure [8,22,41]. Therefore, it is highly possible that the strengthened protein-solvent interactions are an important determinant for the increased overall conformational flexibility of pSHMT. In fact, when compared to mSHMT, pSHMT has a higher abundance of the polar uncharged residues (35.2% vs. 34.5%) and is more negatively charged at the neutral pH (with net charges of −9 vs. −7), in particular with more negative charges exposed on the solvent accessible surface [20]. These are likely responsible for the strengthened protein-solvent interactions of pSHMT because (i) the side chains of the polar uncharged residues are exposed with a high probability on the solvent-accessible protein surface [42] and, (ii) water molecules interact more favorably with the electro-negative surface than with the electro-positive surface of the protein [24,43,44].

Our ED analyses reveal that the largest-amplitude collective motions along the first eigenvector characterize the overall loosening/widening of the dimer in pSHMT while the loosening of the upper part and the contraction/tightening of the lower part of the dimer in mSHMT (Figure 5). This partially explains why pSHMT has a less compact packing and why its two monomers experienced relative position shifts to a larger extent compared to mSHMT during simulations. It has been suggested that decreasing the number and strength of the inter-monomer interactions is an important adaptation strategy of the cold-adapted multimeric enzymes [45]. For pSHMT and mSHMT, the inter-monomer NCIC are 24404 ± 1495 and 25629 ± 1091, respectively, the estimated inter-monomer van der Waals interaction energies are −1886.0 ± 124.1 and −2003.1 ± 81.6 kJ/mol, respectively, and the electrostatic interaction energies are −1496.3 ± 205.7 and −2561.0 ± 242.2 kJ/mol, respectively. Therefore, pSHMT has fewer and weaker inter-monomer interactions/forces than mSHMT, thus explaining the observed trend of the inter-monomer dissociation motions and the considerably enhanced fluctuation amplitudes of the loops located at or close to the inter-monomer interfaces of pSHMT. Nevertheless, we did not observe the dissociation between the monomer-A and -B during the simulations of pSHMT, implying that the high flexibility at the protein-protein interfaces may in turn benefit the protein polymerization at low temperatures, which has been suggested for the microtubule assembly of the cold-adapted β-tubulins [46]. Furthermore, we also observed that, although the fluctuation modes (i.e., amplitudes and directions of the fluctuations) of the structural segments that participate in the formation of the active-site cavity are more complicated in pSHMT than in mSHMT, the net effects of their collective motions are to enlarge and shrink the active-site cavities of pSHMT and mSHMT, respectively. For enzymes, the dynamic variations of the substrate-binding cavity/pocket along the first few eigenvectors generally manifest as the opening/closing, enlarging/shrinking, or twisting of the whole or parts of the cavity, which could be related to the substrate recognition, binding, and orientation and the product release [47]. In the case of pSHMT, because the enlargement of the active-site cavity was observed along the most significant motion mode, such enlargement may be related to its broad substrate specificity [17]; in addition, the increased complexity of the fluctuation modes of the cavity-forming segments may be more advantageous in modulating the thermodynamics and kinetics of the enzyme-substrate interactions compared to mSHMT.

The comparison of the constructed FELs reveals that the FEL of pSHMT covers a larger area of the essential subspace, features a more rugged energy surface, and has a lower minimum free energy level than that of mSHMT. It should be noted that the multiple-replica MD simulations cannot sample the unfolding process but merely achieved as complete a sampling as possible of a protein close to its native state; therefore, our constructed FELs are incomplete and merely represent a significant portion of the near native-state energy landscapes. Nevertheless, the characteristic differences between the FELs of pSHMT and mSHMT are compatible with those between the idealized funnel-like FEL models proposed by Feller et al. for explaining the stability-flexibility-activity relationships in extremophilic enzymes [48].

In the funnel-like FEL model, the width, roughness/ruggedness, and height/depth of the funnel represent the conformational entropy, the degree of the conformational diversity, and the energetic stabilization of the native state versus the unfolded states (i.e., the folding free energy), respectively [49,50]. Since the psychrophilic enzyme features the decreased number and strength of intra-molecular forces/interactions compared to the corresponding mesophilic/thermophilic homologues, it should have a lower structural/thermal stability, higher degree of conformational freedom, and more conformational states/substates; therefore, the idealized psychrophilic FEL was described to have a shallower depth, wider width, and more rugged surface characterized by more minima/basins, in particular at the bottom of the funneled FEL [48]. 

In fact, such characteristic differences between the FELs of differently temperature-adapted homologous enzymes arise from their different molecular flexibilities. From a structural point of view, the flexibility can be described as a concept related to the amplitude of the structural fluctuations on a specific timescale and at a given temperature, whereas from the perspective of the physicochemical principles, the flexibility can be defined as a concept related to the ability of crossing energy barriers that separate the adjacent basins/minima (or of converting between different states/substates) distributed over the FEL. The low barriers, which mean a small enthalpy increase accompanied by a relatively large entropy increase for interconversion/fluctuation between states/substates [51,52,53], are primarily dictated by the high conformational flexibility, with the local flexibility dominating the fast exploration of different substates (within different local minima) of comparable energy levels, whereas the global flexibility governing the slow large-scale collective motions involved in the transition between different states (within different basins) [41,54]. Thus, for the differently temperature-adapted SHMTs, the following scenario could be considered: the increased local flexibility of pSHMT lowers the barriers that separates the local minima and, hence, accelerates the conversion between different substates; the increased global flexibility, together with the enhanced inter-monomer mobility, lowers the barriers between the adjacent basins, thus allowing pSHMT to convert between different states, ultimately leading to a larger conformational entropy and richer conformational diversity. According to the conformational selection mechanism for ligand recognition [55], it is reasonable to consider that the enhanced conformational diversity increases the probability for pSHMT to sample the states/substates competent for association with diverse substrates; upon substrate binding, the increased flexibility of pSHMT allows it to cost less energy to climb the activation energy barrier compared to mSHMT, thus ensuring the high catalytic activity at low temperatures.

## 4. Materials and Methods

### 4.1. Structural Preparation

The crystal structures of mSHMT and pSHMT were obtained from the Protein Data Bank (PDB; http://www.rcsb.org (accessed on 1 September 2020)), with PDB IDs of 1DFO (2.4 Å) [18] and 4P3M (1.85 Å) [19], respectively. For 1DFO (mSHMT), since it contains the atomic coordinates of two functional dimers, one of them formed by the chains C and D was removed and only the one formed by the chains A and B was retained; the atomic coordinates of the missing residue Met1 in the chain A was recovered by taking the corresponding coordinates in the chain B. For 4P3M (pSHMT), there are 46 and 33 missing residues (located in the loops) in the chains A and B, respectively, whose atomic coordinates were modeled using the SWISS-MODEL server [56]. The structure-based sequence alignment was obtained using the Dali server (http://ekhidna2.biocenter.helsinki.fi/dali (accessed on 5 September 2020)) [57] and visualized using ESPript 3.0 (http://espript.ibcp.fr/ESPript/cgi-bin/ESPript.cgi (accessed on 5 September 2020)) [58].

### 4.2. MD Simulations

All MD simulations were performed using the GROMACS 5.1.4 software package [59] with the AMBER99SB-ILDN force field [60]. The two prepared dimer structures were individually solvated with the TIP3P water model [61] in a dodecahedron box, with a minimum solute-box wall distance of 1.0 nm. Counter-ions were added to neutralize the net charge of the protein-solvent systems while reaching a 0.15 M NaCl concentration. An initial steepest descent energy minimization was carried out for each system until no significant energy change could be detected, and this was followed by a series of 100-ps MD runs in the NVT ensemble at 300 K, with the protein heavy atoms restrained by decreasing harmonic potential force constants of 1000, 100, 10, and 0 kJ/mol/nm^2^ to effectively “soak” the solute into the solvent. Before the production MD run, a 400-ps MD run was performed in the NPT ensemble without any restraint. Finally, to improve the conformational sampling of each system, ten independent 100-ns production MD runs were performed, with each of them being initialized with different atomic initial velocities generated by the Maxwell distribution at 300 K. In the production MD simulations, the leapfrog integrator was used with a 2-fs time step and all bonds constrained using the LINear Constraint Solver (LINCS) algorithm [62]; system coordinates were saved every 10 ps; the modified Berendsen (V-rescale) [63] thermostat was used to control the system temperature at 300 K with a time constant of 0.1 ps; the pressure was maintained at 1 atm using the Parrinello-Rahman barostat [64] with a time constant of 2.0 ps; the long range electrostatic interactions were treated using the Particle Mesh Ewald (PME) [65] algorithm, with a real-space cut-off of 1.0 nm, Fourier grid spacing of 0.12 nm, and interpolation order of 4; the van der Waals (vdW) interactions were treated using the Lennard-Jones potential with a cut-off distance of 1.0 nm. 

### 4.3. Analysis Methods

For each production MD trajectory/replica, the time-dependent C_α_ root mean square deviation (RMSD) relative to the starting structure was calculated using the GROMACS tool ‘gmx rms’ to evaluate the stability of the structures during the simulation. For each system, the equilibrated portions of the 10 independent replicas were concatenated into a single joined trajectory, for which the degree of sampling convergence was assessed by calculating the cosine contents of the first two eigenvectors derived from the essential dynamics (ED) analysis. The value of cosine content ranges from 0 to 1, with a high value (i.e., close to 1) meaning that the largest-amplitude protein motions resemble random diffusion and hence the sampling is insufficient, while a low value (i.e., close to 0) signifying an adequate sampling convergence [66]. The per-residue C_α_ root mean square fluctuation (RMSF), which is used as an index of the conformational flexibility, was computed using the GROMACS tool ‘gmx rmsf’. Several structural/geometrical parameters, including the solvent accessible surface areas (SASA), the number of close inter-atomic contacts (NCIC), radius of gyration (Rg), and the number of hydrogen bonds (NHB), were calculated by the GROMACS tools ’gmx sasa’, ‘gmx mindist’, ‘gmx gyrate’, and ‘gmx hbond’, respectively.

The ED method, also called the principal component analysis (PCA), is a powerful tool for reducing the number of dimensions needed to describe the molecular motions by filtering the observed dynamics from the largest to smallest spatial scales [67,68]. This method is based on the diagonalization of the covariance matrix built from atomic fluctuations in a MD trajectory, with the obtained eigenvectors and corresponding eigenvalues representing the motion modes in the conformational space and the atomic fluctuation amplitudes along the corresponding modes, respectively. The C_α_ covariance matrices were built and diagonalized using the GROMACS tool “gmx covar’, followed by the projection of the trajectory onto the eigenvector with the tool ‘gmx anaeig’. The largest-amplitude motion mode along the first eigenvector was visualized using the porcupine plot, which were obtained using the ‘modevectors.py’ script (available from: https://pymolwiki.org/index.php/Modevectors (accessed on 12 October 2020)) with the two extremes extracted from the eigenvector projection as the input. The eigenvectors 1 and 2 were chosen as the reaction coordinates to reconstruct the two-dimensional free energy landscape (FEL) using the probability density function *F*(*s*) = −*k*_B_*T*ln(*N_i_/N_max_*), where *k*_B_ is Boltzmann’s constant, T is the simulation temperature, *N_i_* is the probability of finding the system in state *i*, and *N_max_* is the probability of the most probable state.

## 5. Conclusions

In this work, the cold-adaptation mechanism of SHMT was investigated by performing μs-scale multiple-replica MD simulations on the psychrophilic pSHMT and mesophilic mSHMT followed by a series of comparative analyses in terms of the dynamics, structural properties, and FEL. The dynamics-related analyses reveal that, when compared to mSHMT, pSHMT exhibits significantly enhanced global structural fluctuations and inter-monomer mobility, increased overall flexibility, and considerably enhanced local flexibility involving the surface loops, active sites, and cofactor sites. Based on these differences, it can be concluded that pSHMT likely adopts the global-flexibility mechanism to adapt to the cold environment, with the considerably increased local flexibility playing roles in either lowering the activation free energy or modulating the kinetics and thermodynamics of enzyme-substrate interactions. The comparison between the calculated structural/geometrical parameters indicate that pSHMT is more loosely packed and structurally unstable than mSHMT; although this can be accounted for by the reduced and weakened intra-molecular interactions/forces of pSHMT, we consider that the strengthened interactions between pSHMT and the solvent play an essential role in reducing and weakening the intra-molecular forces and, consequently, it could be concluded that optimizing the protein-solvent interactions is the main adaptation strategy adopted by pSHMT to enhance its structural fluctuations and conformational flexibility. Since the entrance of the active-site cavity is located at the inter-monomer interfaces, along the most significant motion mode of pSHMT the observed trend of the inter-monomer dissociation, in conjunction with the enlargement of the active-site cavity, may explain its broad substrate specificity. Furthermore, the loosening of the association between the two monomers is also responsible for the considerably enhanced mobility in the regions located at or close to the inter-monomer interfaces, and hence, could be considered as another cold-adaptation strategy of the dimeric pSHMT. Finally, the comparison between the constructed FELs indicates that pSHMT has a larger conformational entropy, richer conformational diversity, and lower thermostability than mSHMT. Since the FEL provides a picture that integrates the thermodynamics, kinetics, and dynamics of a protein, the observed differences between the constructed FELs of the two enzymes explain well why the increased flexibility lowers the thermostability (or structural stability) and enriches the conformational diversity of pSHMT and, further, why pSHMT is capable of maintaining its broad substrate specificity and high catalytic activity at low temperatures. 

## Figures and Tables

**Table 1:** Average values and SDs (in parentheses) of structural/geometrical parameters calculated from the single joined equilibrium MD trajectories of mSHMT and pSHMT.

				NHB ^d^	
	SASA ^a^ (Å^2^)	NCIC ^b^	Rg ^c^ (Å)	Intra-Protein	Protein-Solvent
mSHMT	29,103 (400)	985,559 (4580)	27.4 (0.13)	693 (1.3)	1468 (43)
pSHMT	31,577 (632)	957,507 (5087)	28.2 (0.29)	659 (1.4)	1476 (34)

^a^ Solvent accessible surface area. SASA is the surface area of a protein that is accessible to a solvent probe with a 1.4 Å radius. ^b^ Number of close inter-atomic contacts. A close contact is considered to exist if the distance between two atoms is less than 6 Å. ^c^ Radius of gyration. Rg is defined as the root mean square distance from each atom of the protein to their centroid. ^d^ Number of hydrogen bonds. A hydrogen bond is considered to exist if the distance between the hydrogen donor and acceptor atoms is less than 3.5 Å and the angle of donor-hydrogen-acceptor is greater than 120°.

**Table 2:** RMSF average values and SDs (in parentheses) of the active-site components of the two monomers in mSHMT and pSHMT.

Components	mSHMT		pSHMT	
	Monomer-A (nm)	Monomer-B (nm)	Monomer-A (nm)	Monomer-B (nm)
Floor	0.069 (0.009)	0.063 (0.008)	0.085 (0.014)	0.095 (0.017)
Walls	0.085 (0.021)	0.082 (0.019)	0.156 (0.069)	0.116 (0.014)
Roof	0.102 (0.018)	0.094 (0.011)	0.539 (0.149)	0.374 (0.182)

## Data Availability

All data are contained within the article or its supplementary materials as Figures or Tables.

## Supplementary Materials

The following are available online at https://www.mdpi.com/1422-0067/22/4/1781/s1, Figure S1: Structural based sequence alignment of mSHMT and pSHMT; Figure S2: Time evolution of the C_α_ RMSD values of one monomer after least-squares fitting to the same or another monomer in the starting dimeric structure during the multiple-replica MD simulations; Table S1: Standard deviations (SDs) of the RMSD means and cosine contents (CCs) of the first two eigenvectors (Eig.1 and Eig.2) calculated based on the 10-100 ns trajectories of the 10 independent MD simulation replicas and the single joined equilibrium trajectories (only for CCs).
