# Supplementary material for: Exploring the Cold-Adaptation Mechanism of Serine Hydroxymethyltransferase by Comparative Molecular Dynamics Simulations
Source: Int J Mol Sci. 2021 Feb 11;22(4):1781. doi: 10.3390/ijms22041781 (PMC7916883; doi:10.3390/ijms22041781)
Supplement: Supplementary file 1 [file ijms-22-01781-s001.pdf]

Supplementary Materials

Exploring the cold-adaptation mechanism of serine hydroxymethyltransferase by comparative molecular dynamics simulations

Zhi-Bi Zhang [1,2,†], Yuan-Ling Xia [1,†], Guang-Heng Dong [1], Yun-Xin Fu [1,3,*] and Shu-Qun Liu [1,*]

1 State Key Laboratory for Conservation and Utilization of Bio-Resources in Yunnan & School of Life Sciences, Yunnan University, Kunming 650091, China; zhangzhibi@kmmu.edu.cn (Z.-B.Z.); xiayl@ynu.edu.cn (Y.-L.X.); dgh@mail.ynu.edu.cn (G.-H.D.)

2 Yunnan Key Laboratory of Stem Cell and Regenerative Medicine & Biomedical Engineering Research Center, Kunming Medical University, Kunming 650500, China

3 Human Genetics Center and Division of Biostatistics, School of Public Health, the University of Texas Health Science Center, Houston 77030, USA

* Correspondence: Yunxin.Fu@uth.tmc.edu (Y.-X.F.); shuqunliu@ynu.edu.cn (S.-Q.L.)

† These authors contributed equally to this work.

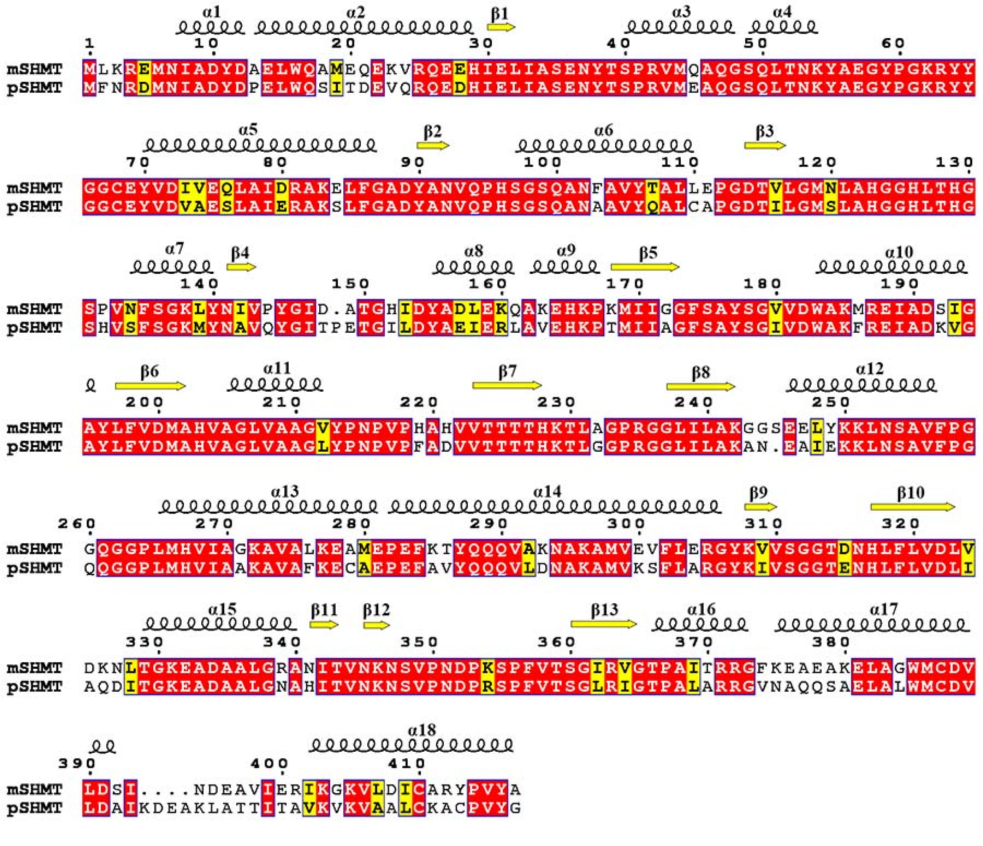

Figure S1. Structural based sequence alignment of mSHMT and pSHMT. Identical residues are white on red background and similar residues are black on yellow background. The amino acid residue numbering and the assignment of the regular secondary structural elements are according to the crystal structure of mSHMT (PDB ID: 1DFO), with black spirals and orange arrows representing α-helices and β-strands, respectively. Residue deletion is denoted by '·' .

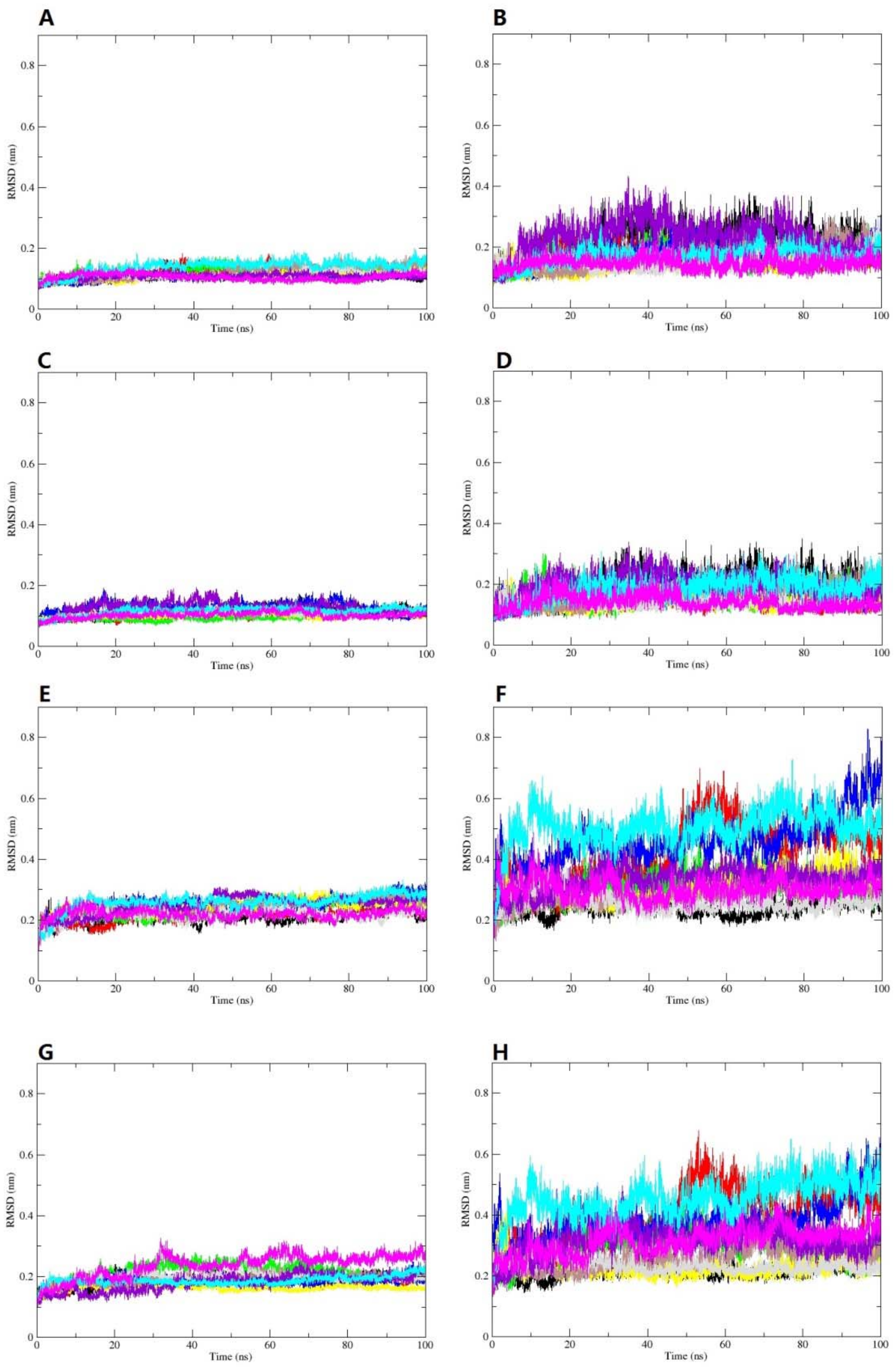

Figure S2. Time evolution of the Cα RMSD values of one monomer after least-squares fitting to the same or another monomer in the starting dimeric structure during the multiple-replica MD simulations. (A) and (B) mSHMT's RMSD curves of the monomer-A after least-squares fitting to the monomer-A and monomer-B, respectively. (C) and (D) mSHMT's RMSD curves of the monomer-B after least-squares fitting to the monomer-B and monomer-A, respectively. (E) and (F) pSHMT's RMSD curves of the monomer-A after least-squares fitting to the monomer-A and monomer-B, respectively. (G) and (H) pSHMT's RMSD curves of monomer-B after least-squares fitting to the monomer-B and monomer-A, respectively.

Table S1. Standard deviations (SDs) of the RMSD means and cosine contents (CCs) of the first two eigenvectors (Eig.1 and Eig.2) calculated based on the 10-100 ns trajectories of the 10 independent MD simulation replicas and the single joined equilibrium trajectories (only for CCs).

Replicas	SDs		CCs			
	mSHMT	pSHMT	mSHMT		pSHMT	
			Eig.1	Eig.2	Eig.1	Eig.2
1	0.0100	0.0336	0.1202	0.0085	0.0039	0.0122
2	0.0124	0.0176	0.0115	0.0002	0.0010	0.0076
3	0.0105	0.0183	0.0413	0.0797	0.0199	0.0011
4	0.0104	0.0133	0.0039	0.0340	0.0001	0.0159
5	0.0184	0.0177	0.0562	0.0051	0.0037	0.0165
6	0.0092	0.0105	0.0306	0.0159	0.0022	0.0196
7	0.0164	0.0209	0.0339	0.1259	0.7011	0.1577
8	0.0116	0.0186	0.0003	0.0055	0.0032	0.0007
9	0.0080	0.0179	0.2147	0.0735	0.0344	0.0002
10	0.0197	0.0141	0.0045	0.0568	0.0144	0.0067
t-test[a]/Joined[b]	p-value = 0.014		0.0175	0.0108	0.0050	0.0052

[a] One-sided t-test was performed to determine whether the SDs of the RMSD means calculated from pSHMT replicas are significantly higher than those from mSHMT replicas. [b] "Joined" represents cosine contents of the first two eigenvectors calculated from the single joined equilibrium trajectories.
